# The Pandemic Affection of Grippe

**Published:** 1918-11

**Authors:** 


**The Pandemic Affection of Grippe.** By H. VIOLLE. *La Presse Médicale*, October 10, 1918.

The morbidity and mortality caused by the grippe have frightened some minds. Some have pretended it was a form of plague; some have called it " dengue "; but none of the terms cover the real disease. This epidemic came from the East and infested first the populations of the Mediterranean coast line, Spain, France and Italy. Fairly mild at first, it became most virulent. The incubation is very short, — sometimes less than 24 hours; the intoxication is extremely rapid. It is this rapidity that often causes death by toxemia.

The causal factor is not known yet, but so far it seems that the grippe microbe is a filtering virus, extremely thin and mobile, and passing through the finest filters. If it were possible, it would also be traced in the urines, but what is absolutely certain is that contamination is propagated through the nasal and pharyngeal expectorations. Nasal mucus, droplets and the like are disseminated about as the patient coughs or sneezes, and eventually find their way to the mucus of the upper respiratory organ.

Because of this, agglomerations in closed halls, theaters, cinemas, large shops, railway carriages, restaurants, etc., should be avoided.

On the contrary, air, light, and warmth are unfavorable grounds for the microbe.

The grippe is in itself benign enough, though it causes an extraordinary prostration of the whole being to the extent of making all the organs a prey to the microbes.

Because of this sideration the antibacterial agents of the tissues can not react and are swamped in the mass invasion of the virus.

In the grippe, there are no lesions in the intestine, but the respiratory tract is affected slightly but definitely. This facilitates the migration of bacteria, such as pneumococci, streptococci, Pfeiffer bacilli, etc. Then there will be such affections as pulmonary congestion or intense broncho-pulmonary troubles, or even veritable purulent melting of the lungs, hemorrhagic pleurisy or any of the possible localizations created by a toxigenous and pyogenous virulent microbe.

The treatment consists of isolation, tepid baths, milk diet, quinine and aspirin, tanin diuretics and light laxatives. The vital point is to prevent the complications coming chiefly from the action of the pneumococcus. Such a serum as that prepared by Nicolle and Truche might be helpful; the serum, of course, being in accordance with the germ found in the sputum.

In other parts of the world complications have been due to the streptococcus. For this form an anti-streptococcic serum might answer well. In intricate forms a compound serum would be the most recommendable.

Injections with the patient's own blood have given very good results indeed. This can be repeated several days in succession without injury.

Among other precautions the hospital personnel must wear gowns and masks,—a mask made of a piece of gauze folded in four.

The ideal condition would be to segregate each patient, even if only by mobile partitions.

Violle and Défossine, when they experimented vaccinating ward attendants, had very good results indeed, but these experiments, being few, should not yet be taken as conclusive.

At home the prophylactic measures will be just the same. However, it is well to emphasize that Dr. Roux of the Institut Pasteur insists on the wearing of the mask, which was one of the surest means of defense in the epidemic of pneumonic plague in Manchuria.

